# A reducing demand for tertiary hospital‐based gender affirming care in Victoria, Australia

**DOI:** 10.1111/imj.70265

**Published:** 2025-12-13

**Authors:** Sarah Lum, Donna Eade, David Colon Cabrera, Riki Lane, Gurvinder Kalra, Ken C. Pang, Ada S. Cheung

**Affiliations:** ^1^ Gender Clinic, Department of Endocrinology Austin Health Melbourne Victoria Australia; ^2^ Royal Children's Hospital Gender Service Melbourne Victoria Australia; ^3^ Monash Health Gender Clinic Melbourne Australia; ^4^ Murdoch Children's Research Institute Melbourne Victoria Australia; ^5^ Department of Paediatrics The University of Melbourne Melbourne Victoria Australia; ^6^ Trans Health Research Group, Department of Medicine The University of Melbourne Melbourne Victoria Australia

**Keywords:** transgender persons, gender clinic, health service delivery

## Abstract

This study presents a retrospective audit of new referrals to three tertiary gender clinics in Victoria, Australia, from 2020 to 2024. It finds a plateau and slight reduction in referrals, in contrast to previous increases over the preceding decade. The reduction may stem from a shift to primary and community‐based services. These findings suggest decentralising care could ease strain on tertiary clinics while maintaining access, informing models for the delivery of genderaffirming care in Australia.

There have been reports of an exponential rise in demand for gender affirming care in many countries around the world, particularly during the period 2010 to 2018.^1^, ^2^, ^3^, ^4^ Reasons for the increasing demand are unclear and difficult to investigate, but hypotheses include an increase in visibility, access to information, availability of gender affirming health services and reduction in societal stigma.^1^, ^2^, ^3^, ^4^ Recent data suggest that between 2017 and 2019, the number of individuals identifying as trans or gender diverse (trans) may have stabilised,^5^, ^6^ but this is certainly not universal, with some international studies reporting ongoing increases.^7^ Demand for gender affirming surgery appears to have continued to increase up until 2021.^8^

Accurate data on the demand for gender affirming services are important given that there is much political debate regarding the provision of gender affirming care to trans young people but, more importantly, to ensure adequate service delivery to meet the demand and healthcare needs of trans people. We aimed to audit the three tertiary referral hospital gender clinics in the state of Victoria, Australia, to assess the number of new referrals annually. Based on emerging data,^5^ we hypothesised that there would be a plateau in new referral numbers annually.

This was a retrospective audit of new referral numbers over 4 years from 1 January 2020 to 31 December 2024 at the only three tertiary referral hospitals (Austin Health Gender Clinic servicing adults, Monash Health Gender Clinic servicing adults and Royal Children's Hospital Gender Service servicing children and adolescents <16 years) in Victoria, Australia. The audit was approved by the Austin Health Human Research Ethics Committee (HREC/106309/Austin‐2024) and the Royal Children's Hospital HREC (36323). Dates were chosen as Austin Health Gender Clinic commenced in December 2019 with full calendar year data available from 2020 to 2024. Data for the same time period were also obtained from Monash Health Gender Clinic and Royal Children's Hospital. New referral numbers in aggregate (deidentified) for each year were extracted from existing electronic medical records at Austin Health (a clinic predominantly Medicare Benefits Schedule funded 2020 to 2024) and a clinical database at the Royal Children's Hospital. For Monash Health, referral numbers were extracted from routine reportable data that are sent to the Department of Health.

Analyses were performed in R version 4.3.1 using the base stats package. Variability in total annual referrals for all three hospitals was assessed with a Pearson chi‐squared goodness‐of‐fit test. Longitudinal trends were evaluated by ordinary least‐squares regression of Referrals on Year; the regression slope (β), 95% confidence interval, coefficient *P*‐value and *R* ^2^ are reported. Unless otherwise specified, tests were two‐sided with α = 0.05; exact *P*‐values were reported.

New referrals made to each hospital clinic are shown in Figure 1. Annual total new referrals were 999 (2020), 1440 (2021), 1322 (2022), 1162 (2023) and 944 (2024). Totals varied significantly across years in a goodness‐of‐fit test against a uniform distribution (χ^2^ = 150.3, *P* < 0.0001). Between 2020 and 2024, there was no significant linear trend (slope −38.8 referrals/year, 95% CI: –272.1 to 194.5; *P* = 0.63; *R* ^2^ = 0.09). Analysis restricted to 2021–2024 showed referrals declined significantly year‐on‐year (slope − 164.8 referrals/year, 95% CI: –233 to −96.6; *P* = 0.009; *R* ^2^ = 0.98). Pairwise contrasts were directionally concordant: the increase from 2020 to 2021 (χ^2^ = 58.9, *P* < 0.0001) and the decline from 2021 to 2024 (χ^2^ = 77.1, *P* < 0.0001) were significant.

**Figure 1:**
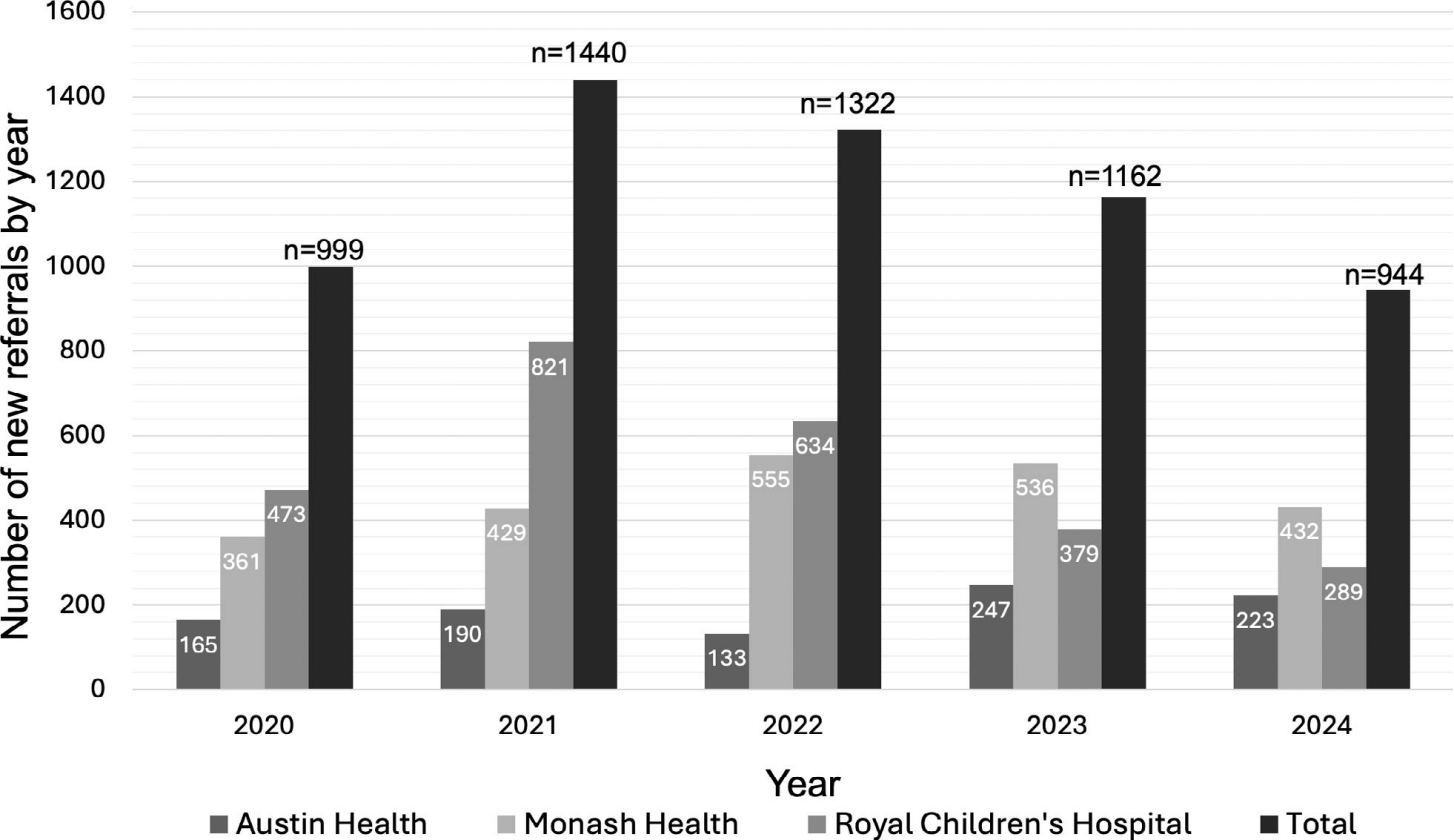
Tertiary Hospital New Gender Clinic Referrals received from 1 January 2020 to 31 December 2024.

## Discussion

Overall, the total number of new referral numbers for gender affirming care across tertiary hospitals in Victoria peaked in 2021 and then reduced significantly thereafter with 2024 being the lowest year observed. The reduction in referrals after 2021 may be explained by increasing services in primary care and in the community.

Across Victoria, gender affirming medical care is provided through three tertiary hospital services and a distributed network of primary‐care, community health and private‐sector providers (including sexual health and endocrinology clinics). There has been a concerted effort to shift gender affirming care to primary care (general practitioners and community health) with the development of a state government‐funded training programme for health professionals in transgender health in 2018. Moreover, the community‐based Orygen Trans and Gender Diverse Service commenced in 2023 to serve youth aged 12–25 to minimise pressure on tertiary hospital waiting lists. There are also multiple private primary care clinics across Victoria that provide assessment and initiation of gender affirming hormones for post‐pubertal adolescents and adults. While this study was not a probability sample and did not capture all community‐based trans services, data were available for all specialist tertiary gender services in the state, which had previously seen an exponential rise in demand.^1^, ^9^

Overall, the early signs of reduction in referral numbers to tertiary hospital gender clinics in our state are novel. Further studies incorporating data from primary and community care settings would serve to provide valuable insight. Education of health professionals and provision of non‐tertiary services in the community may be alleviating access and demand on specialist tertiary gender services. This may be an effective strategy for other healthcare services that face ongoing increases in referral numbers and allow for optimisation of resource allocation. Further research incorporating qualitative measures of patient satisfaction and outcomes to capture the broader impact of shifting care pathways on trans people is needed. Ultimately, research across diverse settings in other jurisdictions globally will clarify if this reduction represents a shift.

## Data Availability

The data that support the findings of this study are available from the corresponding author upon reasonable request.
